# The Usage of Histamine Type 1 Receptor Antagonist and Risk of Dementia in the Elderly: A Nationwide Cohort Study

**DOI:** 10.3389/fnagi.2022.811494

**Published:** 2022-03-18

**Authors:** Chuan-Chi Yang, Wu-Chien Chien, Chi-Hsiang Chung, Chung-Yu Lai, Nian-Sheng Tzeng

**Affiliations:** ^1^Department of Psychiatry, Taoyuan Armed Forces General Hospital, Taoyuan, Taiwan; ^2^Department of Psychiatry, Taoyuan Armed Forces General Hospital, Hsinchu Branch, Hsinchu City, Taiwan; ^3^Department of Psychiatry, Tri-Service General Hospital, School of Medicine, National Defense Medical Center, Taipei, Taiwan; ^4^Department of Medical Research, Tri-Service General Hospital, National Defense Medical Center, Taipei, Taiwan; ^5^School of Public Health, National Defense Medical Center, Taipei, Taiwan; ^6^Graduate Institute of Life Sciences, National Defense Medical Center, Taipei, Taiwan; ^7^Taiwanese Injury Prevention and Safety Promotion Association, Taipei, Taiwan; ^8^Graduate Institute of Aerospace and Undersea Medicine, National Defense Medical Center, Taipei, Taiwan; ^9^Student Counseling Center, National Defense Medical Center, Taipei, Taiwan

**Keywords:** histamine type 1 receptor antagonist, dementia, National Health Insurance Research Database, nationwide cohort study, neurotranmitters

## Abstract

**Background:**

The histamine type 1 receptor antagonist (H1RA) has been commonly used. This study aimed to examine the association between the usage of H1RA and the risk of dementia.

**Methods:**

A total of 8,986 H1RA users aged ≥50 and 26,958 controls matched a ratio of 1:3 for age, sex, and comorbidity, were selected between January 1, and December 31, 2000, from Taiwan’s National Health Insurance Research Database. Fine and Gray’s survival analysis (competing with mortality) was used to compare the risk of developing dementia during a 15-year follow-up period (2000–2015).

**Results:**

In general, the H1RA usage was not significantly associated with dementia (adjusted subdistribution hazard ratio [SHR] = 1.025, 95% confidence interval [CI] = 0.883–1.297, *p* = 0.274) for the H1RA cohort. However, a differential risk was found among the groups at risk. The patients with the usage of H1RA aged ≥65 years (adjusted SHR: 1.782, 95% CI = 1.368–2.168, *p* < 0.001) were associated with a higher risk of dementia, in comparison to the control groups. Furthermore, the patients with the usage of H1RA that were male, or had more comorbidities, were also associated with an increased risk of dementia.

**Conclusion:**

The usage of H1RA was associated with the risk of developing dementia in the patients aged ≥ 65 years.

## Introduction

Dementia is one of the most devastating age-related neuropsychiatric diseases, which denotes a wide range of cognitive decline and behavioral disturbances, with a lifetime prevalence of about 5–7% in the global population (Prince et al., 2013). In Taiwan, the prevalence of dementia for patients aged ≥65 years was 4–8% (Sun et al., 2014), with an increasing impact on the patients, their caregivers, and the community (Tzeng et al., 2015, 2017b; Wang et al., 2018). Several modifiable risks and protective variables that could be potentially addressed so as to prevent or delay the onset of dementia (Tzeng et al., 2016, 2018a, 2019a, 2020). As a result, efforts will be required to identify the risk variables and then decrease any potential exposure to those risk factors.

Histamine, a neurotransmitter, plays a role in several physiological functions in the human body, such as inflammation, gastric acid secretion, and vessels dilation (Simons and Simons, 2011), with four subtypes of receptors, being, H1, H2, H3, and H4 receptors (H1R–H4R). Several histamine receptor agonists and antagonists have been developed for the histaminergic system (Tiligada et al., 2011). H1 histamine receptor antagonists (H1RA) are frequently used in clinical practice to treat allergic conditions such as asthma, conjunctivitis, food allergies, rhinitis, and atopic dermatitis (Theoharides and Stewart, 2016). Activation of the histamine H1 receptor, which is expressed widely in the body, led to either inositol phosphate accumulation or intracellular calcium mobilization while solely H2 receptors are involved with the stimulation of gastric secretion. Both H1 and H2 receptors mediate opposing physiological and pharmacological effects on the cardiovascular system and in the lungs (Parsons and Ganellin, 2006). Histamine H3 receptors are presynaptic auto-receptors that constrain histamine synthesis and are released in the histaminergic neurons in the CNS and modulate the release of other neurotransmitters in the CNS and periphery (Leurs et al., 2005). The H4 receptor, which is preferentially expressed in the immune cells, has been demonstrated homology with the H3 receptor and several H3 agonists and antagonists also bind to the H4 receptor (Jablonowski et al., 2004).

The histamine receptors (H1, H2, H3, and H4) are all the G-protein-coupled receptor members. H1, H2, and H3 were known to have spontaneous activity in the absence of an agonist, known as constitutive activity. Therefore, the antagonists can be classified as inverse agonists under these circumstances (Morisset et al., 2000). H1RA are classified into the first- or second-generation antihistamines. First-generation H1RAs, such as diphenhydramine, chlorpheniramine, and ketotifen, have a sedating effect because of the passage into the brain (Sharma et al., 2015). Second-generation H1RAs, such as cetirizine, fexofenadine, and loratadine, are less likely used for sedation, since they are substrates for the blood–brain barrier P-glycoprotein, an efflux pump, that could decrease their concentration in the CNS (Hu et al., 2015). Second generation H1RAs also have a higher specificity and affinity for the peripheral H1 receptor than the first generation H1RA that has the antihistamine and anticholinergic effects (Kavosh and Khan, 2011). H1 antihistamines are also known to be used in psychiatric settings as sedatives. anxiety, dystonia, and extrapyramidal syndrome, for their anticholinergic effects (Oken, 1995). Given that the potential adverse effects of the antihistamine with anticholinergic effects, there is growing interest in the association between antihistamine usage and the risk of dementia.

The anticholinergic effect was distressing due to their effects on the cognition problems, including sedation, drowsiness, delirium, and memory impairments, especially in the elderly (Cai et al., 2013; Liu et al., 2020). Previous studies have revealed that drugs with an anticholinergic effect could worsen the cognitive and physical function in the elderly and further underscored its association between dementias (Ruxton et al., 2015; Zheng et al., 2021). While the H2 histamine receptor antagonists (H2RA) also have been depicted to have an anticholinergic effect which has a potential risk to impair the cognitive function (Chew et al., 2008). One prospective longitudinal study with a 5-year follow-up found an association between the H2RA usage and the incidence of cognitive impairment in a cohort of elderly African-Americans (Boustani et al., 2007). A retrospective population-based cohort study of individuals aged 65 years and older revealed a significantly increased risk of dementia associated with the usage of H2RA (Chen et al., 2020). Three other longitudinal studies, however, did not support the prospective or retrospective association between H2RA and the risk of dementia (Zandi et al., 2002; Hanlon et al., 2004; Gray et al., 2011). Therefore, a nationwide, population-based, longitudinal study is still needed to clarify the association between the H1RA exposure and dementia. In the present study, a matched cohort study was carried out and the Taiwan National Health Insurance Research Dataset (NHIRD) was used to clarify if there was an association between H1 antihistamine usage and dementia in patients aged over 50 years during a 15-year follow-up period.

## Materials and Methods

### Data Sources

The National health insurance (NHI) Program, a Taiwanese single-payer compulsory social insurance plan which commenced from 1995, covers 97% of the health care providers enrolled in Taiwan, which includes approximately 23 million beneficiaries (Ho Chan, 2010). Several previous studies described the details of this program (Tzeng et al., 2016, 2017a,c, 2018b,c,d, 2019b,c,d,e; Chien et al., 2017a,b; Chang et al., 2018; Chao et al., 2018, 2019; Chu et al., 2018; Kao et al., 2018; Yang et al., 2018; Tseng et al., 2019). The NHIRD stores comprehensive and detailed clinical records of more than 99% of the population, including diagnoses coded by the International Classification of Diseases, 9th Revision, Clinical Modification (ICD-9-CM), for research purposes. Several studies have ascertained the accuracy and validity of the diagnoses, such as myocardial infarction (Cheng et al., 2015), diabetes (Lin et al., 2005), oral cancer (Li-Ting et al., 2014), and stroke (Cheng et al., 2011), in the NHIRD. The database also recorded all prescriptions dispensed from 1995 to the present, including drug name and amount dispensed. The Taiwan National Health Research Institute established a subset of the NHIRD, the Longitudinal Health Insurance Database (LHID) dataset, which contains health claims and registration for 2,000,000 randomly sampled patients from the total beneficiaries registered in the NHI program. There were no statistically significant differences in the claim data between the 2,000,000 sampled individuals and the population.

### Study Design and Sampled Participants

This is retrospective matched-cohort research using the LHID between January 1, 2000, and December 31, 2015. Each patient aged 50 years or older was required to receive the treatment with first- and second generation H1RA in the inpatient and outpatient settings within the first one-year study period. A 1:3 sex-, age-, and insurance premium-matched, index year- matched, location-matched, level of care-matched, controls were randomly selected for each patient with H1RA. The exclusion criteria for the cohorts were unknown sex, subjects diagnosed with dementia or receiving H1RA before the index date, or <50 years old during the study period. The index date was defined as the time when the individuals received their first H1RA within the one-year study period. Notably, self-reported over the counter (OTC) H1RA usage and duration was not available because of the study design. Since antihistamines are contraindicated in the following conditions, individuals diagnosed with glaucoma, bladder neck obstruction, benign hypertrophy of prostate with urinary obstruction and other lower urinary tract symptoms, peptic ulcer with esophagus stricture, pyloric stricture and duodenal stricture were also excluded (all these ICD-9-CM codes are as listed in Supplementary Table 1). Cumulative exposure was categorized by the estimated duration of the H1RA usage based on the total defined daily dose (DDD) per package prescribed before any dementia diagnosis, as 1–364 days, 365–1,459 days, >1,460 days with cut-points based on the clinical interpretability.

### Outcomes

All individuals in the study and control group were followed from the index date until the diagnosis of dementia, death, withdrawal from the NHI program, or the end of 2015. Patients diagnosed with dementia were identified by the ICD-9-CM codes with Alzheimer dementia (AD), vascular dementia (VaD), and other degenerative dementia (all of these ICD-9-CM codes listed in Supplementary Table 1).

### Covariates

The covariates included the sociodemographic and comorbidities. Sociodemographic characteristics included sex, age (50–64, ≥65 years), season of index date, regions of residence, urbanization levels, and levels of medical care. The reason for enrolling the individuals aged >50 is that this cohort study has a 15-year follow-up. We could therefore analyze the long-term influences of H1RA on the risk for dementia. The monthly insured premiums have been divided into three categories in New Taiwan Dollars [NT$]: <18,000, 18,000–34,999, ≥35,000. The urbanization level of residence has been classified according to the indicators of the development of a city, such as population, public health, economic, and environmental changes. Level 1 represented a region with a population greater than 1,250,000, and the level 4 region, having a population of <149,999, was the most rural region (Chang et al., 2014).

The Charlson comorbidity index (CCI) is one of the most widely used comorbidity indexes (Charlson et al., 1987; de Groot et al., 2003). The CCI consists of 22 conditions (Charlson et al., 1994), including diabetes, cerebrovascular disease, and hemiplegia (stroke) (Mayeux and Stern, 2012) which were mostly associated with AD. CCI was used to quantify the comorbidities since it could predict the clinical outcome for patients who may have simultaneous chronic conditions.

### Statistical Analysis

The SPSS software version 22 (SPSS Inc., Chicago, IL, United States) was used to conduct the statistical analyses. The Pearson *chi-square* test was used for the analysis of the categorical data. Continuous variables presented as the mean [±standard deviations (SD)], were analyzed using the two-sample *t*-test. To investigate the risk of AD, VaD, and other dementia for patients with and without H1RA treatment, the Fine and Gray’s model was used to conduct the competing risk analysis by the sub-distribution hazard ratios (SHRs) and 95% confidence intervals (CIs), adjusting for sociodemographic characteristics, and comorbidities. The Kaplan–Meier method was used to determine the difference in the risk of dementia for the study and control groups using the log-rank test. The subgroup analysis on the incidence of dementia were conducted by dividing them into two sub-types, first-generation H1RA versus second-generation H1RA. A *p* value <0.05 was considered statistically significant. Sensitivity analyses were conducted excluding all dementia cases occurring within the first two years and five years of the start of the research.

## Results

### Baseline Characteristics of the Study Population

**FIGURE 1:**
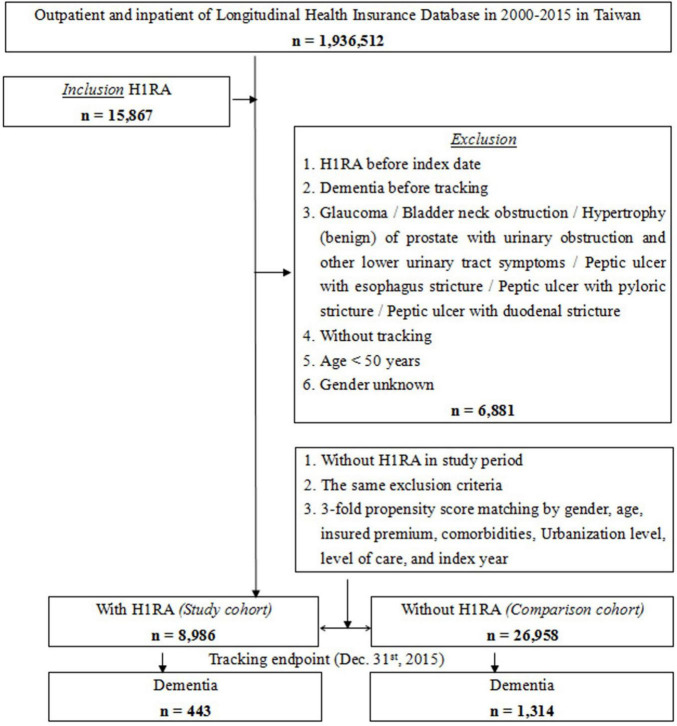
The flowchart of study sample selection.

A total of 8,986 patients who had been administered with H1RAs and 26,958 control group patients matched for sex, age, comorbidity, insured premium, level of care, urbanization level, and index year were enlisted from the follow-up period (Figure 1). The mean age (±SD) of the patients with H1RA was 59.65 ± 24.12 years, and 54.11% were male (Table 1). Sociodemographic and clinical features of patients of the two groups are as displayed and no significant differences were found between both groups in age, sex, level of care, CCI score, and monthly insurance premiums.

**TABLE 1:** Characteristics of study in the baseline.

H1RA	With		Without		*P*
Variables	*n*	%	*n*	%	
**Total**	8,986	25.00	26,958	75.00	
**Gender**					0.999
Male	4,862	54.11	14,586	54.11	
Female	4,124	45.89	12,372	45.89	
**Age (years)**	59.65 ± 24.12		59.63 ± 24.07		0.946
**Age group (years)**					0.999
50–64	4,862	54.11	14,586	54.11	
≥65	4,124	45.89	12,372	45.89	
**Insured premium (NT$)**					0.989
<18,000	6,672	74.25	20,009	74.22	
18,000–34,999	1,452	16.16	4,372	16.22	
≥35,000	862	9.59	2,577	9.56	
**CCI_R group**					0.938
0	3,450	38.39	10,372	38.47	
1–3	5,116	56.93	15,350	56.94	
≥4	420	4.67	1,236	4.58	
**Season**					0.999
Spring (March–May)	2,214	24.64	6,642	24.64	
Summer (June–August)	2,336	26.00	7,008	26.00	
Autumn (September–November)	2,220	24.71	6,660	24.71	
Winter (December–February)	2,216	24.66	6,648	24.66	
**Location**					0.963
Northern Taiwan	2,563	28.52	7,680	28.49	
Middle Taiwan	2,215	24.65	6,688	24.81	
Southern Taiwan	2,420	26.93	7,259	26.93	
Eastern Taiwan	1,467	16.33	4,412	16.37	
Outlets islands	321	3.57	919	3.41	
**Urbanization level**					0.562
1 (The highest)	3,411	37.96	10,106	37.49	
2	3,510	39.06	10,498	38.94	
3	779	8.67	2,335	8.66	
4 (The lowest)	1,286	14.31	4,019	14.91	
**Level of care**					0.997
Hospital center	3,428	38.15	10,272	38.10	
Regional hospital	3,020	33.61	9,067	33.63	
Local hospital	2,538	28.24	7,619	28.26	

*P, Chi-square/Fisher exact test on category variables and t-test on continue variables. NT$, New Taiwan Dollars, CCI_R, Charlson Comorbidity Index, dementia removed.*

### The Association Between H1RA and Dementia in the Elderly

During the 15-year follow-up period, dementia developed in 443 in the study cohort (*N* = 8,986) and 1,314 in the control group (*N* = 26,958) developed dementia (507.19 vs 497.37 per 100,000 person-years). Table 2 manifested the results of the investigation on the association between the H1RA usage and the dementia risk, which were analyzed by the Fine and Gray’s competing risk model. After adjusting for sex, age, CCI, geographical region, and the urbanization level of residence, level of care, and monthly income, the H1RA usage was not associated with an elevation in the overall risk of dementia (adjusted HR = 1.025 [95% CI: 0.883–1.297], *p* = 0.274) However, the risk of dementia was associated with the H1RA users aged ≥65 (adjusted HR = 1.782 [95% CI: 1.368–2.168], *p* ≤ 0.001) (Table 2). In addition, the H1RA users being male (adjusted SHR = 1.889 [95% CI: 1.187–2.284], *p* ≤ 0.001), with a CCI score of >4 (adjusted SHR = 1.352 [95% CI: 1.173–1.579], *p* ≤ 0.001), and receiving medical care from the regional hospitals (adjusted HR = 1.430 [95% CI: 1.110–2.517], *p* ≤ 0.001) or medical centers (adjusted SHR = 1.488 [95% CI: 1.124–2.525], *p* ≤ 0.001), were associated with an increased risk of developing dementia. Furthermore, Supplementary Table 2 shows that H1RA users aged ≥65 were associated with the different types of dementia, including AD (adjusted SHR = 1.811 [95% CI: 1.427–2.295], *p* < 0.001), VaD (adjusted SHR = 1.663 [95% CI: 1.210–1.979], *p* < 0.001), and other dementias (adjusted SHR = 1.506 [95% CI: 1.095–1.832], *p* < 0.001).

**TABLE 2:** Factors of dementia by using Cox regression with/without Fine and Gray’s competing risk model.

	Competing risk in the model							
Variables	Crude SHR	95% CI	95% CI	*P*	Adjusted SHR	95% CI	95% CI	*P*
**H1RA**								
Without	Reference				Reference			
With	1.129	0.549	2.901	0.672	1.025	0.883	1.297	0.274
**Gender**								
Male	1.917	1.210	2.345	<0.001	1.889	1.187	2.284	<0.001
Female	Reference				Reference			
**Age group (year)**								
50–64	Reference				Reference			
≥65	1.988	1.469	2.486	<0.001	1.782	1.368	2.168	<0.001
**Insured premium (NT$)**								
<18,000	Reference				Reference			
18,000–34,999	0.962	0.608	1.510	0.401	0.939	0.579	1.462	0.435
≥35,000	0.907	0.511	1.439	0.477	0.880	0.482	1.380	0.501
**CCI_R group**								
0	Reference				Reference			
1–3	1.209	1.030	1.492	0.021	1.189	0.997	1.402	0.054
≥4	1.428	1.258	1.688	<0.001	1.352	1.173	1.579	<0.001
**Season**								
Spring	Reference				Reference			
Summer	1.065	0.709	1.548	0.462	1.061	0.692	1.531	0.475
Autumn	1.138	0.803	1.703	0.352	1.129	0.757	1.684	0.367
Winter	1.142	0.811	1.711	0.348	1.137	0.761	1.699	0.362
**Location**								
Northern Taiwan	Reference				Multicolline arity with urbanization level			
Middle Taiwan	0.923	0.578	1.352	0.420	Multicolline arity with urbanization level			
Southern Taiwan	0.984	0.624	1.397	0.387	Multicolline arity with urbanization level			
Eastern Taiwan	0.863	0.488	1.262	0.612	Multicolline arity with urbanization level			
Outlets islands	0.557	0.178	3.972	0.883	Multicolline arity with urbanization level			
**Urbanization level**								
1 (The highest)	1.431	0.928	1.942	0.250	1.309	0.813	1.872	0.272
2	1.309	0.815	1.925	0.299	1.238	0.792	1.824	0.331
3	1.104	0.612	1.720	0.348	1.031	0.519	1.680	0.399
4 (The lowest)	Reference				Reference			
**Level of care**								
Hospital center	1.697	1.301	2.630	<0.001	1.488	1.124	2.525	<0.001
Regional hospital	1.632	1.225	2.591	<0.001	1.430	1.110	2.517	<0.001
Local hospital	Reference				Reference			

*P, Chi-square/Fisher exact test on category variables and t-test on continue variables; H1RA, histamine receptor type 1 antagonist; CI, confidence interval, adjusted SHR, adjusted sub-distribution hazard ratio, adjusted variables listed in the table, NT$, New Taiwan Dollars, CCI_R, Charlson Comorbidity Index, dementia removed.*

### Kaplan–Meier Model for the Cumulative Incidence of Dementia

**FIGURE 2:**
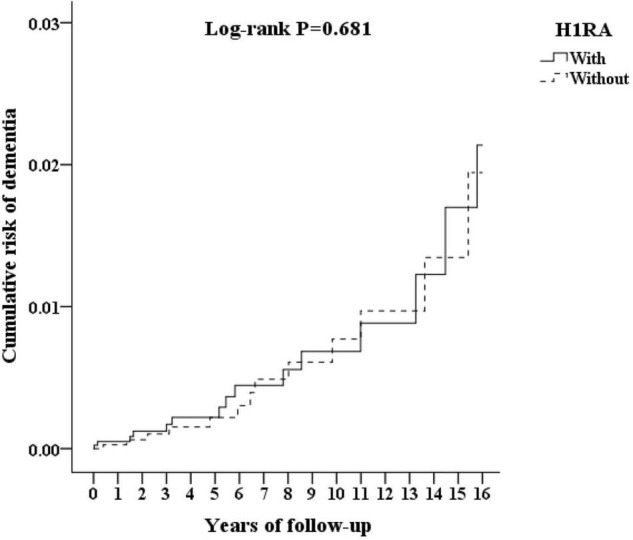
Kaplan–Meier for cumulative risk of dementia aged 50 and over stratified by H1RA (histamine type 1 antagonist) with log-rank test.

The cumulative incidences of the primary end point are as shown in Figure 2 indicating the cumulative risk of dementia between the H1RA cohort and the control group. According to the Kaplan–Meier method, the incidence rate of dementia was not associated with the H1RA usage (log-rank test *P* = 0.681).

### Use of First- and Second-Generation Antihistamines and the Risk of Dementia

Table 3 illustrates the H1RA subgroups analysis for the first- (G1) and second- (G2) generation H1RA (*N* = 6,975 vs 2,011) and the doses of H1RA exposure. While 345 subject (3.83%) developed dementia in the subgroup with G1 H1RA, 98 (1.0%) developed dementia in those using G2 H1RA within the follow-up period. The adjusted HR of the G1 and G2 H1RA subgroup in the development of dementia were 1.029 (95% CI: 0.888–1.304, *p* = 0.260) and 1.013 (95% CI: 0.872–1.265, *p* = 0.279), respectively. In addition, the adjusted SHR of AD, VaD, and other dementias were 1.028 (*p* = 0.279), 1.025 (*p* = 0.258), and 1.026 (*p* = 0.275), respectively. The H1RA was not associated with the increased risk of dementia in different cumulative dosages. The duration of H1RA exposure, analyzed with cumulative DDD, was not associated with an increased risk of dementia. The adjusted HR of H1RA exposure longer than four years in the incidence of dementia were 1.035 (95% CI: 0.906–1.384, *p* = 0.249).

**TABLE 3:** Factors of dementia in different model by using Cox regression with/without Fine and Gray’s competing risk model.

Model	H1RA					Competing risk in the model			
	Subgroups	Populations	Events	PYs	Rate (per 10^5^ PYs)	Adjusted HR	95% CI	95% CI	*P*
**Model 1**	Without	26,958	1,314	264,188.40	497.37	Reference			
H1RA subgroup	With	8,986	443	87,343.93	507.19	1.025	0.883	1.297	0.274
	G1 H1RA	6,975	345	67,784.05	508.97	1.029	0.888	1.304	0.260
	G2 H1RA	2,011	98	19,559.88	501.03	1.013	0.872	1.265	0.279
**Model 2**	Without, 0	26,958	1,314	264,188.40	497.37	Reference			
DDD	1–364	2,731	135	26,549.41	508.49	1.028	0.886	1.328	0.271
	365–1,459	3,865	189	37,568.23	503.08	1.017	0.881	1.359	0.284
	≥1,460	2,390	119	23,226.29	512.35	1.035	0.906	1.384	0.249

*P, Chi-square/Fisher exact test on category variables and t-test on continue variables; H1RA, histamine type 1 antagonist; PYs, Person-years; G1 H1RA, First-Generation 1 histamine type 1 antagonist, G2 H1RA, Second-Generation histamine type 1 antagonist; Adjusted HR, Adjusted sub-distribution hazard ratio: Adjusted for the variables listed in Table 1. CI, confidence interval; DDD, daily dose defined.*

### The Interaction Term Analysis Between Age and H1RA Usage

Supplementary Table 3 shows the subgroup analysis for the factors of dementia using the Cox regression and Fine and Gray’s competing risk model. The p-values of the interaction term analysis of Age × H1RA usage was 0.001 in the non-competing risk model and <0.001 in the competing risk model.

## Discussion

In this population-based retrospective cohort study, we described the patients aged older than 50 with H1RA usage and found that the H1RA was not associated with the elevated risk of dementia. The risk of different types of dementia was not significantly associated with H1RA usage. A subgroup analysis showed that the patients with H1RA exposure were associated with the risk of dementia, including males, aged ≥65 years, scores of CCI, and the care from the medical center and regional hospitals, in comparison to the control group. The adjusted SHR for patients aged ≥65 years was 1.782 (95% CI: 1.368–2.168, *p* ≤ 0.001) in comparison to the patients aged 50–64 years (Table 2). Namely, the patients aged ≥65 years in the H1RA cohort exhibited a 1.7-fold increased risk of developing dementia. The interaction term analysis shows that there are interactions between age and H1RA usage (Supplementary Table 3). Two sensitivity analyses conducted to ameliorate the influences from a protopathic bias showed that even after excluding the patients diagnosed with dementia within the first two years and five years, the H1RA cohorts were still not associated with an increased risk of dementia.

Our findings are similar to other studies, including a nested case-control study of 58,769 persons ≥55 years investigating the association between the risk of dementia and different type of drugs with the anticholinergics effect, including antihistamine, that was reported as having no significantly increased risks of dementia from its lowest to highest exposure levels categorized by the total standardized daily drug dose (Coupland et al., 2019). The heaviest level of anticholinergic exposure equivalent included 50 mg of diphenhydramine or 75 mg hydroxyzine each day for longer than three years (Gray et al., 2015). However, cumulative exposure calculated by the total standardized daily drug with heterogenous properties rather than a specific drug type was conducted in the study. In addition, although the second-generation H1RA is much more selective for the peripheral H1 receptor than the first-generation H1RA, and therefore the H1 antihistamines of choice in the clinical setting, cetirizine has reported several conflicting results in previous studies of its effect on behavioral performance (Theunissen et al., 2004; Vacchiano et al., 2008), and working memory (van Ruitenbeek et al., 2008, 2010). While the association between the second generation H1RA and dementia required further investigation with the advanced study design and analysis, and it is not associated with the risk of dementia in the present study.

Patients aged ≥65 years with H1RA were associated with an elevated risk of dementia, in comparison to the patients aged 50–64 years, in the present study. Previous research has concluded that aging itself is a risk factor of dementia development (Liu et al., 1995; Sun et al., 2014). We might also attribute the risk of dementia to aging rather than the influence of H1RA itself. Nonetheless, antihistamines are a typical treatment in older patients with allergic rhinitis, conjunctivitis, and other allergic skin conditions (Hansen et al., 2005).

Over the counter sleep aids containing first-generation H1RA with anticholinergic effects, mostly diphenhydramine and doxylamine, used by community-dwelling older adults as well as patients with AD to promote sleep was also very common. Notably, a previous study had suggested a bidirectional relationship between AD and sleep deprivation, which may increase accumulation of the brain amyloid-β causing wakefulness and alternation of sleep patterns (Ju et al., 2014). According to Beers’ criteria, drugs with potent anticholinergic effects are inappropriate for older people (American Geriatrics Society Beers Criteria Update Expert Panel, 2019). Many older adults, however, especially those with chronic medical conditions causing sleep disturbance, turn to OTC drugs with H1RA to improve sleep without paying attention to their potential risks (Abraham et al., 2017). One retrospective study of patients aged 75 years or older diagnosed with dementia in Japan, based on claims data between 2010 and 2013, found a total of 12,658 participants in the study, with 8,272 (65.3 %) of them receiving first-generation H1RA (Maeda et al., 2018). The finding in this study might well serve as a reminder for the clinicians caring for those who have been exposed to H1RA about the potential disadvantage and risk of dementia in individuals aged ≥65 years.

Furthermore, the increased risk of dementia was associated with males, the CCI scores, and the medical care from the medical center and regional hospital. This might support the previous findings that dementias are prevalent in patients with more medical comorbidities (Chen et al., 2017) or even need a higher medical attention (Kaczynski et al., 2019). Males with H1RA usage were associated with a higher risk of dementia, in comparison with women with H1RA usage. Previous studies that have investigated gender differences in the risk of dementia showed contentious results. Females have been shown to have a higher incidence of dementia than in males in several studies, especially AD (Hebert et al., 2001; Kukull et al., 2002; Miech et al., 2002), whereas others have shown no difference (Bachman et al., 1993; Rocca et al., 1998; Mielke, 2018). A population-based study suggested there was no gender differences in the risk of AD up to a higher age until aged older than 90 years and concluded males have a higher incidence of vascular dementia than women in all age groups (Ruitenberg et al., 2001). A study investigating the gender differences in the incidence of dementia, based on a large population-based cohort, suggested a cardiovascular factor, but not a gender factor independently, should be considered since the fact that selective survival of males with a healthier cardiovascular risk profile might occur and result in a lower propensity to dementia (Chêne et al., 2015). Overall, a better understanding of the gender differences along with other risk factors or mechanisms could lead to an improvement of the treatment for both genders with dementias.

Although the histaminergic system has been thought to play a role in the neurotransmission in the central nervous system, its level of activity in the neuropsychiatric diseases needs further investigation. Through the histamine receptor, it contributes to the regulation of wakefulness, cognition, and the circadian rhythm (Haas et al., 2008; Thakkar, 2011). Mitochondrial dysfunction has been proposed as one of the possible pathophysiological mechanisms involved in AD (Swerdlow, 2018). It has been reported that histamine receptors might play a role in the mitochondrial dysfunction and causes oxidative stress along with mitochondrial damage (Rocha et al., 2016). Previous studies have found changes of the histaminergic neurons in the tuberomammillary nucleus in the early stages of AD (Braak et al., 1993; Schneider et al., 1997). Significant histaminergic neurons losses have been found in patients with Alzheimer (Motawaj et al., 2010) and decreased H1 histamine receptor binding as well as histamine content might play an important role in their cognitive impairment (Higuchi et al., 2000). In vascular dementia, a lack of histaminergic transmission in the brain has also been reported (Stasiak et al., 2011). Contrariwise, it has been found that an increased histamine content in the brains and blood serum of patients with AD (Cacabelos et al., 1989, 1992). Furthermore, previous studies have shown that the H3 histamine receptor can inhibit the release of histamine, norepinephrine, dopamine, and acetylcholine in the brain (Passani et al., 2004; Gemkow et al., 2009). Thus, H3RA have been demonstrated as a potential therapeutic target for the treatment in several clinical disorders and cognitive deficits in neuropsychiatric diseases (Sadek et al., 2016). For example, novel H3R antagonist/inverse agonist, CEP-26401, has been displayed to elicit amnesic effects in rodent models and have a potential utility in cognitive disorder, attention disorder, and adjunctive treatment for schizophrenia (Raddatz et al., 2012). Overall, future research could be focused on the histamine as well as neurotransmission systems and their mechanism of mitochondrial dysfunctions in the brains of patients with dementias.

Several limitations of the present study should be noted. First, the information regarding dementia severity, staging, impact on the caregiver burden, genetic, psychosocial, laboratory and image data, and environmental factors were not included in the NHIRD database. Instead of the direct medical records or the interview data, they are retrospective and rely on the ICD-9-CM codes and therefore misdiagnosis-related errors should be considered. In particular, the NHIRD claim data lacking the clinical details regarding the indication for antihistamine usage or detailed information about the inflammatory process were not included in the present study, given the interactions of inflammatory mediators and the pathophysiological mechanisms of dementias could not be ignored. This is the common limitation for studies using administrative claims datasets, including the NHIRD. We have tried to focus on certain events that would be available. Second, it is possible that OTC H1RA could have been used by both groups before the enrollment of the two cohorts since the OTC H1RA usage was not included in the NHIRD. Furthermore, although the records of the usage of antihistamine, based on the prescription records, were recorded in the NHIRD, the medication compliance and the rate of refills could not be assessed. DDD as a methodology instead of precise medication dosage was conducted because of the lack of daily dosage and supply information. Third, other unmeasured and residual confounding, including other medications with an anticholinergic effect, could introduce bias in this study. The results might not reflect the fact that many different healthcare professionals have been involved in patient care, so the measurement of the risk factors and outcomes throughout the database would probably be less accurate and consistent than that obtained with a prospective cohort study design. In particular, the increased risk found in the higher CCI score groups could be related to the overall worsening of the health condition and polypharmacy is common in the older population with multimorbidity. Fourth, although H1RA users aged ≥65 years exhibited an increased risk of developing dementia in contrast to those aged 50–64 years, differences in the type of dementia or effect of DDD could not be conducted to clarify in the sub-group of patients ≥65 years because of the study design, which included the H1RA cohort as patient above 50 years with a competing risk analysis. Fifth, claims data of NHI beneficiaries, launched since 1995, are initially generated for imbursement rather than for research purposes. Therefore, the content would follow the regulations by the NHI administration and the Computer-Processed Personal Data Protection Law. Diagnostic coding in the NHIRD had been based on codes from ICD-9-CM from 1995 to 2016. ICD-10-CM/PCS was introduced to the NHIRD in 2016. Since ICD11 has been approved for adoption by WHA member states to come into effect in 2022, following approval of the ICD-11 and transition from the ICD-10 to the ICD-11 will be necessary in the future. Sixth, the NHIRD does not contain the data of APOE E4. Therefore, we could not analyze the role of APOE E4 in the association between the H1RA and risk of dementia. Seventh, we must admit that the exhaustive approach in finding the correlations between the covariates and the risk of dementia might limit the value of the present study. One of the methods we tried to minimize this problem is the usage of CCI scores, instead of individual comorbidities. Finally, other types of dementia were found to be proportionately higher than AD and VaD in the present study. However, several community studies have reported that AD is the most common cause of dementia (40–60% in all dementias), followed by VaD (20–30% in all dementias), and mixed or other dementias (7–15%) (Liu et al., 1995; Lin et al., 1998). One possibility for this disparity is that some individuals classified as other degenerative types of dementia, might be AD cases. Another explanation is that the clinicians might encode these types of dementia, which have an insidious onset and progressive course without evidence of previous cerebrovascular events, instead of AD to diagnose the beginning and ongoing dementias in Taiwan. Furthermore, frontotemporal dementia tends to start at a younger age, mostly in people aged 45–65. Since our study included those between 50 and 65 years old, early onset dementia such as frontotemporal dementia, which would be encoded as non-AD in the NHIRD, might be more common in this age group.

## Conclusion

Although H1RA has been found to be associated with cognitive function impairment over the years, a population-based, longitudinal study is required so as to clarify the association between H1RA exposure and dementia. Since all types of dementia may develop progressively and gradually over several years, a longer period of follow-up between the usage of the H1RA drugs and the diagnosis of dementia was crucial. Our study has extended prior research in this area by a considerably large sample and extensive period of follow-up about past H1RA usage. In general, the H1RA usage cohort was not associated with the risk of dementia. However, the usage of H1RA in patients aged ≥65 years was associated with an increased risk of dementia in comparison to the controls. In addition to old age, male gender, and worsening health conditions are significant risk factors for dementia in the H1RA user. The finding could serve as a reminder for clinicians caring for those who have been exposed to H1RA about the potential disadvantage and risk of dementia in older patients to reduce unnecessary H1RAs usage. Future studies might well focus on the role of mitochondrial dysfunction by these drugs and their effects on brain inflammation.

## Data Availability Statement

The original contributions presented in the study are included in the article/Supplementary Material. The datasets on the study population that were obtained from the NHIRD (http://nhird.nhri.org.tw/en/index.html) are maintained in the NHIRD (http://nhird.nhri.org.tw/). The National Health Research Institutes (NHRI) is a nonprofit foundation established by the government. Only citizens of Taiwan who fulfill the requirements for conducting research projects are eligible to apply for the NHIRD. The use of the NHIRD is limited to research purposes only. Applicants must follow the Personal Data Protection Act (https://law.moj.gov.tw/ENG/LawClass/LawAll.aspx?pcode=I0050021) and the related regulations of the National Health Insurance Administration and NHRI, and an agreement must be signed by the applicant and their supervisor upon application submission. All applications are reviewed for approval of data release. Further inquiries can be directed to the corresponding authors.

## Ethics Statement

This study followed the code of ethics of the World Medical Association Declaration of Helsinki and was conducted in accordance with the human research guideline of the Local Ethical Committee. As the dataset encrypted all the identification data, the Institutional Review Board of the Tri-Service General Hospital approved this study and waived the need for individual consent (IRB No. A202105181).

## Author Contributions

All authors made substantial contributions to the conception and design, acquisition of data, or analysis and interpretation of data, took part in drafting the article or critically revising it for important intellectual content, gave final approval of the version to be published, and agreed to be accountable for all aspects of the work.

## Conflict of Interest

The authors declare that the research was conducted in the absence of any commercial or financial relationships that could be construed as a potential conflict of interest.

## Publisher’s Note

All claims expressed in this article are solely those of the authors and do not necessarily represent those of their affiliated organizations, or those of the publisher, the editors and the reviewers. Any product that may be evaluated in this article, or claim that may be made by its manufacturer, is not guaranteed or endorsed by the publisher.

## Abbreviations

AD: Alzheimer dementia
CCI: Charlson comorbidity index
CI: confidence intervals
DDD: defined daily dose
H1RA: H1 histamine receptor antagonist
H2RA: H2 histamine receptor antagonist
H3RA: H3 histamine receptor antagonist
ICD-9-CM: International Classification of Diseases, 9th Revision, Clinical Modification
LHID: Longitudinal Health Insurance Database
NHI: National health insurance
NHIRD: National Health Insurance Research Dataset
NHRI: National Health Research Institute
SD: standard deviations
SHR: subdistribution hazard ratios
VaD: vascular dementia.
