# Supplementary material for: The Usage of Histamine Type 1 Receptor Antagonist and Risk of Dementia in the Elderly: A Nationwide Cohort Study
Source: Front Aging Neurosci. 2022 Mar 18;14:811494. doi: 10.3389/fnagi.2022.811494 (PMC8972197; doi:10.3389/fnagi.2022.811494)
Supplement: Supplementary file 2 [file Table_2.DOCX]

| **Table S2. Comparing the adjusted HR of dementia subgroups by using Cox regression with Fine & Gray's competing risk model** | | | | |
| --- | --- | --- | --- | --- |
| **Age groups (years)** | **≧65 *vs.* 50-64** *(Reference)* | | | |
| **Dementia subgroups** | **Adjusted SHR** | **95% CI** | **95% CI** | ***P*** |
| Overall dementia | 1.782 | 1.368 | 2.168 | <0.001 |
| AD | 1.811 | 1.427 | 2.295 | <0.001 |
| VaD | 1.663 | 1.210 | 1.979 | <0.001 |
| Other dementia | 1.506 | 1.095 | 1.832 | <0.001 |
| **PYs = Person-years; Adjusted SHR = Adjusted Subdivision Hazard ratio: Adjusted for the variables listed in Table 3.; CI = confidence interval** | | | | |
