# Supplementary material for: The Usage of Histamine Type 1 Receptor Antagonist and Risk of Dementia in the Elderly: A Nationwide Cohort Study
Source: Front Aging Neurosci. 2022 Mar 18;14:811494. doi: 10.3389/fnagi.2022.811494 (PMC8972197; doi:10.3389/fnagi.2022.811494)
Supplement: Supplementary file 3 [file Table_3.docx]

| **Table S3. Factors of dementia by using Cox regression with / without Fine & Gray's competing risk model** | | | | | | | | |
| --- | --- | --- | --- | --- | --- | --- | --- | --- |
|  | **No competing risk in the model** | | | | **Competing risk in the model** | | | |
| **Variables** | **Adjusted HR** | **95% CI** | **95% CI** | ***P*** | **Adjusted HR** | **95% CI** | **95% CI** | ***P*** |
| **H1RA** |  |  |  |  |  |  |  |  |
| Without | Reference |  |  |  | Reference |  |  |  |
| With | 1.019 | 0.875 | 1.265 | 0.274 | 1.025 | 0.883 | 1.298 | 0.271 |
| **Gender** |  |  |  |  |  |  |  |  |
| Male | 1.863 | 1.166 | 2.279 | <0.001 | 1.889 | 1.186 | 2.283 | <0.001 |
| Female | Reference |  |  |  | Reference |  |  |  |
| **Age group (years)** |  |  |  |  |  |  |  |  |
| 50-54 | Reference |  |  |  | Reference |  |  |  |
| 55-64 | 1.052 | 0.675 | 1.424 | 0.386 | 1.059 | 0.682 | 1.465 | 0.310 |
| 65-74 | 1.451 | 1.024 | 1.933 | 0.018 | 1.464 | 1.048 | 1.986 | 0.002 |
| 75-84 | 1.760 | 1.341 | 2.128 | <0.001 | 1.774 | 1.365 | 2.160 | <0.001 |
| ≧85 | 1.986 | 1.554 | 2.359 | <0.001 | 1.995 | 1.571 | 2.388 | <0.001 |
| **Insured premium (NT$)** |  |  |  |  |  |  |  |  |
| <18,000 | Reference |  |  |  | Reference |  |  |  |
| 18,000-34,999 | 0.935 | 0.567 | 1.460 | 0.462 | 0.940 | 0.580 | 1.461 | 0.434 |
| ≧35,000 | 0.872 | 0.442 | 1.378 | 0.512 | 0.881 | 0.483 | 1.380 | 0.503 |
| **CCI_R group** |  |  |  |  |  |  |  |  |
| 0 | Reference |  |  |  | Reference |  |  |  |
| 1-3 | 1.168 | 0.984 | 1.398 | 0.068 | 1.189 | 0.996 | 1.401 | 0.055 |
| ≧4 | 1.346 | 1.127 | 1.577 | <0.001 | 1.352 | 1.173 | 1.580 | <0.001 |
| **Season** |  |  |  |  |  |  |  |  |
| Spring | Reference |  |  |  | Reference |  |  |  |
| Summer | 1.060 | 0.689 | 1.528 | 0.481 | 1.062 | 0.692 | 1.530 | 0.475 |
| Autumn | 1.124 | 0.743 | 1.673 | 0.371 | 1.128 | 0.758 | 1.684 | 0.368 |
| Winter | 1.134 | 0.748 | 1.680 | 0.365 | 1.138 | 0.761 | 1.700 | 0.361 |
| **Urbanization level** |  |  |  |  |  |  |  |  |
| 1 (The highest) | 1.300 | 0.811 | 1.868 | 0.289 | 1.309 | 0.814 | 1.872 | 0.272 |
| 2 | 1.234 | 0.790 | 1.825 | 0.341 | 1.239 | 0.791 | 1.824 | 0.335 |
| 3 | 1.028 | 0.512 | 1.675 | 0.403 | 1.031 | 0.519 | 1.680 | 0.399 |
| 4 (The lowest) | Reference |  |  |  | Reference |  |  |  |
| **Level of care** |  |  |  |  |  |  |  |  |
| Hospital center | 1.481 | 1.119 | 2.517 | <0.001 | 1.489 | 1.125 | 2.527 | <0.001 |
| Regional hospital | 1.428 | 1.108 | 2.508 | <0.001 | 1.429 | 1.111 | 2.517 | <0.001 |
| Local hospital | Reference |  |  |  | Reference |  |  |  |
| **HR= hazard ratio, CI = confidence interval, Adjusted HR: Adjusted variables listed in the table** | | | | | | | | |
| **Interaction term (Age × H1RA): *P* = 0.001 (Non-competing risk in the model), *P* < 0.001 (Competing risk in the model)** | | | | | | | | |
